# Asparagine Tautomerization in Glycosyltransferase Catalysis. The Molecular Mechanism of Protein *O*-Fucosyltransferase 1

**DOI:** 10.1021/acscatal.1c01785

**Published:** 2021-07-23

**Authors:** Beatriz Piniello, Erandi Lira-Navarrete, Hideyuki Takeuchi, Megumi Takeuchi, Robert S. Haltiwanger, Ramón Hurtado-Guerrero, Carme Rovira

**Affiliations:** † Departament de Química Inorgànica i Orgànica (Secció de Química Orgànica) and Institut de Química Teòrica i Computacional (IQTCUB), Universitat de Barcelona, Martí i Franquès 1, 08028 Barcelona, Spain; ‡ Institute of Biocomputation and Physics of Complex Systems (BIFI), University of Zaragoza, Mariano Esquillor s/n, Campus Rio Ebro, Edificio I+D, 50018 Zaragoza, Spain; § Department of Biochemistry and Molecular Biology, Complex Carbohydrate Research Center, The University of Georgia, Athens, Georgia 30602, United States; ∥ Fundación ARAID, 50018 Zaragoza, Spain; ⊥ Copenhagen Center for Glycomics, Department of Cellular and Molecular Medicine, University of Copenhagen, 1017 Copenhagen, Denmark; # Institució Catalana de Recerca i Estudis Avançats (ICREA), Passeig Lluís Companys 23, 08010 Barcelona, Spain

**Keywords:** enzymes, O-glycosylation, carbohydrates, glycosyltransferases, quantum mechanics/molecular mechanics, metadynamics

## Abstract

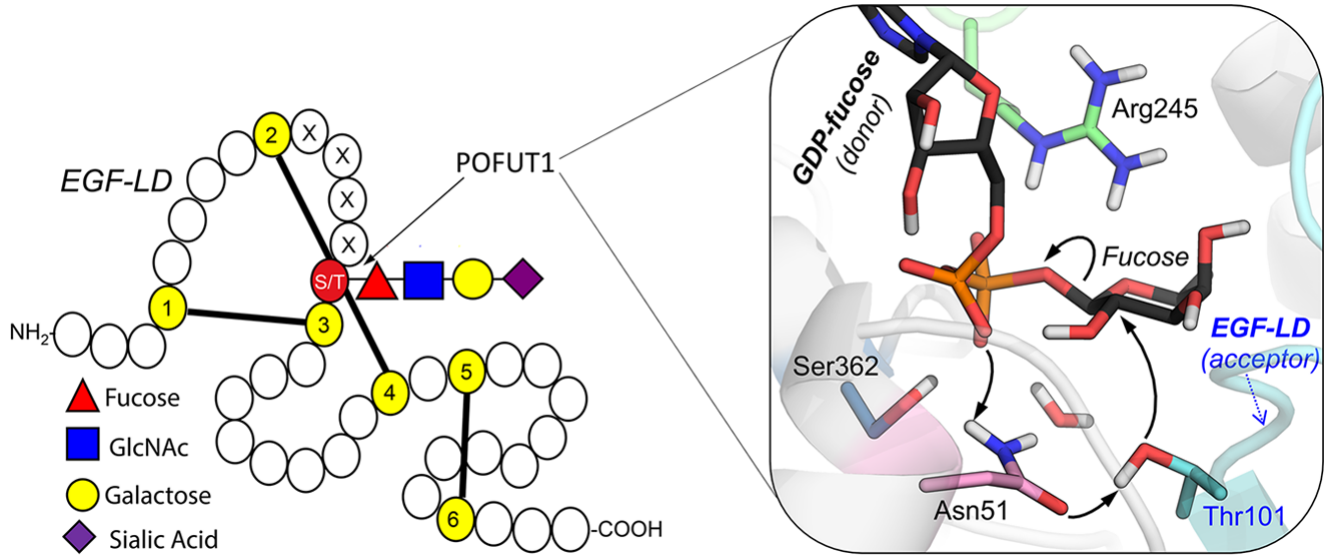

*O*-glycosylation is a post-translational protein modification essential to life. One of the enzymes involved in this process is protein *O*-fucosyltransferase 1 (POFUT1), which fucosylates threonine or serine residues within a specific sequence context of epidermal growth factor-like domains (EGF-LD). Unlike most inverting glycosyltransferases, POFUT1 lacks a basic residue in the active site that could act as a catalytic base to deprotonate the Thr/Ser residue of the EGF-LD acceptor during the chemical reaction. Using quantum mechanics/molecular mechanics (QM/MM) methods on recent crystal structures, as well as mutagenesis experiments, we uncover the enzyme catalytic mechanism, revealing that it involves proton shuttling through an active site asparagine, conserved among species, which undergoes tautomerization. This mechanism is consistent with experimental kinetic analysis of *Caenorhabditis elegans* POFUT1 Asn43 mutants, which ablate enzyme activity even if mutated to Asp, the canonical catalytic base in inverting glycosyltransferases. These results will aid inhibitor development for Notch-associated *O*-glycosylation disorders.

Glycans attached to proteins and lipids are essential molecules in nature; they coat cells in our body and are involved in multiple biological functions, such as regulation of cellular communication, protein folding, and targeting of specific proteins.^1,2^ The formation of the glycosidic linkages in glycans is catalyzed by glycosyltransferases (GTs), highly specific enzymes that utilize an activated donor sugar substrate that contains a substituted phosphate leaving group.^3^ Understanding GTs, not only by elucidating their structures but also by uncovering their molecular mechanisms of action,^4^ is important for controlling their function, boosting future bioengineering applications to synthesize novel glycans.^5^

Fucose is one of the few sugars in metazoans with L stereochemistry, being a common terminal modification on protein and lipid glycans.^6^ There are several fucosyltransferases responsible for attaching a fucosyl unit to glycans, but only two of them catalyze the direct attachment of l-fucose to Ser/Thr residues, an important post-translational modification in higher eukaryotic organisms.^7^ These are protein *O*-fucosyltransferases 1 and 2 (POFUT1 and POFUT2, respectively). POFUT1 is particularly interesting because of its involvement in the Notch signaling pathway (NSP), an essential cell–cell communication pathway conserved in all multicellular animals.^8,9^ The NSP participates in numerous cell-fate decisions both during development and in the mature organisms.^10^ Malfunction of the NSP has been linked to several diseases in humans, such as Dowling–Degos disease,^10^ leukemia,^11^ and colorectal cancer.^12^ POFUT1 catalyzes one of the first steps of the Notch receptors’ maturation, shaping it to be a critical regulator of the Notch cascade.^13,14^ As such, POFUT1 is a potential therapeutic target for the aforementioned Notch-related disorders.

POFUT1 catalyzes the transfer of l-fucose from GDP-β-l-fucose (hereafter fucose and GDP-Fuc, respectively) to the hydroxyl of Ser or Thr with inversion of configuration at the anomeric carbon.^15^ From a structural point of view, POFUT1 belongs to GT family 65 and exhibits a GT-B type fold, consisting of two domains separated by a cleft in which the acceptor substrate binds. The enzyme is located in the endoplasmic reticulum, where it catalyzes the *O*-fucosylation of properly folded epidermal growth factor-like domains (EGF-LDs), small protein domains of about 40 residues that usually contain six or eight conserved cysteine residues (6-Cys, 8-Cys). POFUT1 only *O-*fucosylates acceptor Thr or Ser residues in 6-Cys EGF-LDs, which are present in around 100 human proteins, most notably the four Notch receptors.^16,17^ A consensus sequence for fucosylation of POFUT1 has been identified as C^2^-X-X-X-X-S/T-C^3^, C^2^ and C^3^ being the second and third conserved cysteines, respectively, in the EGF-LDs (Figure 1A).

**Figure 1:**
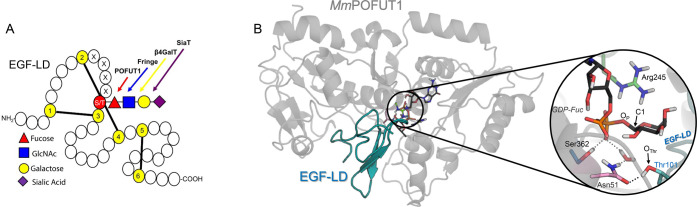
(A) Schematic picture of glycosylated EGF. Disulfide bonding lines are shown as black bold lines. The site of *O*-fucosylation and GlcNAc elongation are indicated by a red triangle and a blue square, respectively. S/T indicates serine/threonine residues. (B) Ternary complex of POFUT1 obtained from QM/MM MD simulations guided by structures PDB 5KY3 and 5KXH (Figure S2). Hydrogen atoms of active site residues have been omitted for clarity, except those attached to heteroatoms.

The first crystallographic structure of POFUT1, as well as the first one among GT65 family members, was solved in 2011 for *Caenorhabditis elegans* POFUT1.^15^ Recently, structures of *Mus musculus*(16) and *Homo sapiens*(10) POFUT1 have also been reported. Analysis of these structures revealed that the active site of POFUT1 differs from that of typical inverting GTs. The most striking feature is that the active site lacks a basic residue that could act as a catalytic base able to deprotonate the Thr nucleophile residue of the acceptor during the expected S_N_2 chemical reaction. Typically an aspartate, glutamate, or a coupled His/Glu plays the role of such a catalytic base (Figure 2A).^3,18^ Interestingly, site-directed mutagenesis studies on *Ce*POFUT1 and *Drosophila melanogaster* POFUT1 showed that two active site residues, an arginine and an asparagine, are essential for enzyme activity.^15^ Both residues are conserved among species (see Supporting Information, SI, Figures S1 and S2).^9,15^ The arginine residue (Arg240 in *Ce*POFUT1/*Dm*POFUT1 or Arg245 in *Mm*POFUT1) forms hydrogen bonds with the GDP phosphate groups and the fucosyl glycosidic oxygen (Figure S2) and this residue was suggested to facilitate the cleavage of the glycosidic bond.^15,16^ The role of the conserved asparagine residue among species (Asn43 in *Ce*POFUT1/*Dm*POFUT1 or Asn51 in *Mm*POFUT1) remains unknown, although it was assumed that it could help to orient the hydroxyl of the Thr/Ser residues of the EGF-LD acceptor in the proper catalytic configuration.

**Figure 2:**
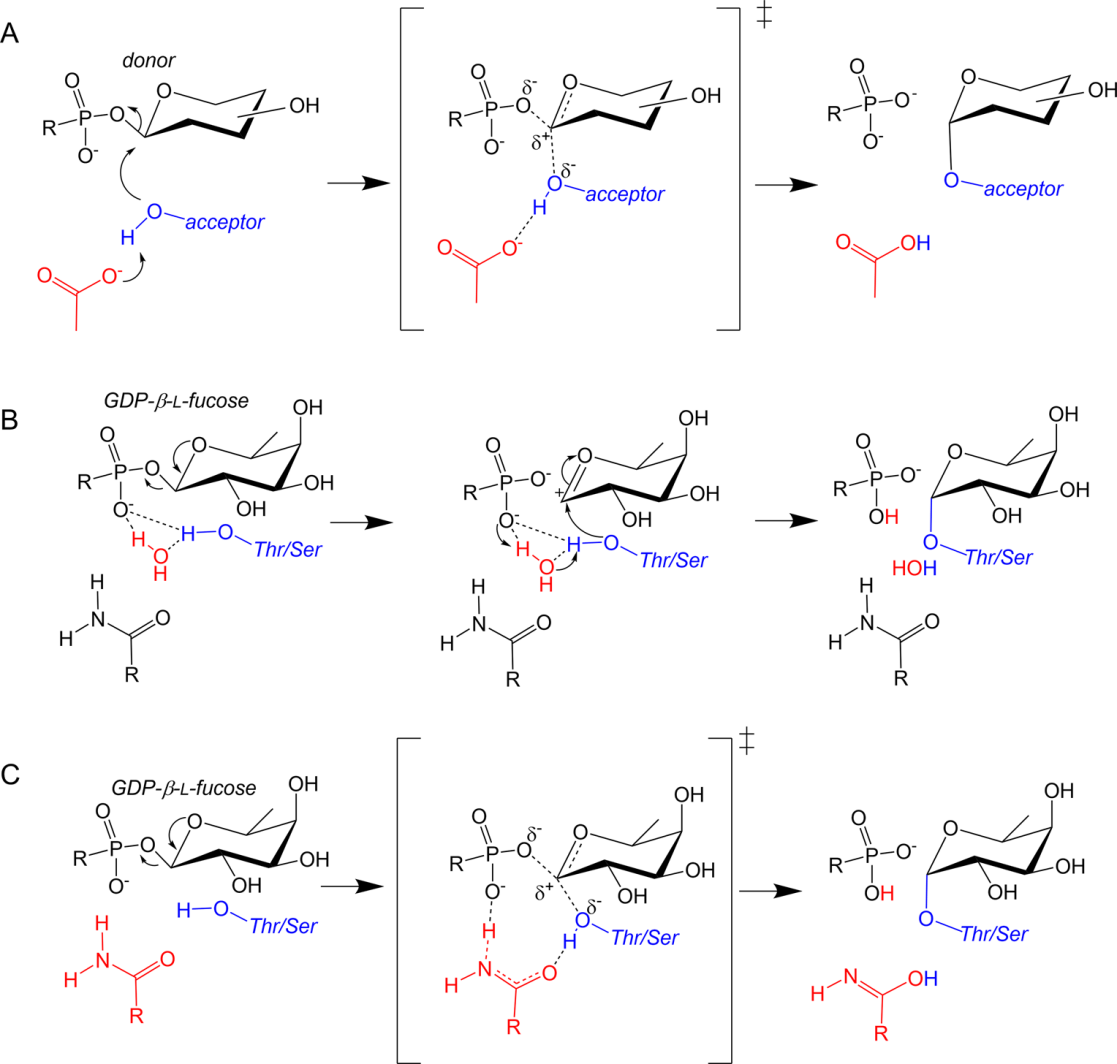
Reaction mechanism of inverting glycosyltransferases. (A) Classical inversion mechanism, in which a carboxylic acid residue acts as general base. The donor sugar is a generic one. (B) Proposed mechanism of POFUT1 on the basis of crystal structures. The transfer of the Thr/Ser acceptor proton to the β-phosphate can take place directly or via a water molecule. (C) Mechanism obtained in this work from QM/MM metadynamics simulations.

The absence of a catalytic base close to the Thr nucleophile residue led to the proposal of a mechanism in which the oxygen of the β-phosphate group directly abstracts the Thr proton (Figure 2B), acting as a catalytic base. Li et al. later suggested that an active site water molecule might assist this process by shuttling the Thr hydroxyl proton toward the β-phosphate oxygen.^16^ The reaction was expected to follow a type of S_N_1 mechanism^15,16^ in which a short-lived oxocarbenium ion would be formed. Interestingly, Li et al.^16^ pointed out that, even though the interaction between the acceptor Thr and Asn51 would not likely facilitate an S_N_2-like reaction, the possibility of a proton transfer role for Asn51 via amide tautomerization could be envisaged, which could be tested by computational approaches.

Here, we uncover the mechanism of catalysis of POFUT1 by computer simulation for the first time, using quantum mechanics/molecular mechanics (QM/MM) metadynamics methods (see SI for details).^19,20^ Metadynamics is an efficient technique for enhancing the sampling in molecular dynamics simulations. One the strengths of metadynamics is that it does not rely on an initial guess of the reaction pathway, and as such the method has been recently used to discover catalytic mechanisms of several glycoside hydrolases (GHs)^21−25^ and GTs.^26−29^ Our simulations show that the Asn51 residue plays an essential catalytic role by mediating proton transfer from the Thr hydroxyl group to the leaving acceptor phosphate. This computational prediction explains previous mutagenesis data showing that enzyme activity is knocked down when Asn51 is mutated to Ala,^15,17^ as well as new kinetic data (this work) showing that the activity cannot be rescued when it is replaced by an aspartate residue as found in classical inverting GTs.

The starting model structure for the simulations was built from the available crystal structures. A ternary complex of wild type POFUT1 with intact donor (GDP-Fuc) and acceptor proteins (EGF-LD) is not available. However, there are two recent high resolution structures^16^ that can be used to build the ternary complex. On one hand, there is a structure of *Mm*POFUT1 in complex with GDP-Fuc and a mutant of the EGF-LD acceptor (the nucleophile Thr101 was replaced by Ala; PDB 5KY3, at 1.53 Å resolution). There is also a structure of *Mm*POFUT1 in complex with GDP and the intact EGF-LD Factor VII acceptor (PDB 5KXH, at 1.33 Å resolution). We used the former structure as a template and reverted the Thr101Ala mutation by taking the Thr101 coordinates from the latter structure (Figure S2).

Molecular dynamics simulations (300 ns), using the Amber suite of programs (see SI for details), showed that the structure of the reconstructed Michaelis complex is stable in time, thus it is a reasonable model to start QM/MM simulations. We found that Thr101 can adopt two different orientations (Figure S3), with one of them displaying the hydroxyl group in an optimal orientation to attack the anomeric carbon of the donor sugar (O_Thr101_···C1 ≈ 4 Å, Figure 3B and Table S1). The simulations also show that there is no basic residue near Thr101 that could deprotonate its hydroxyl during the reaction, as predicted in structural studies.^15,16^ A water molecule remains in the active site in a fluctuating position between Asn51 and the β-phosphate. Interestingly, this floppy water molecule is often near the hydroxyl group of Thr101 (Figure 2B), suggesting that it could mediate proton transfer among the two. The asparagine residue, Asn51, could also play this role, since its amide carbonyl interacts with the Thr101 hydroxyl group and its amide nitrogen interacts with the β-phosphate via the water molecule. Nevertheless, it is not possible to predict the detailed mechanism from the modeled Michaelis complex alone.

**Figure 3:**
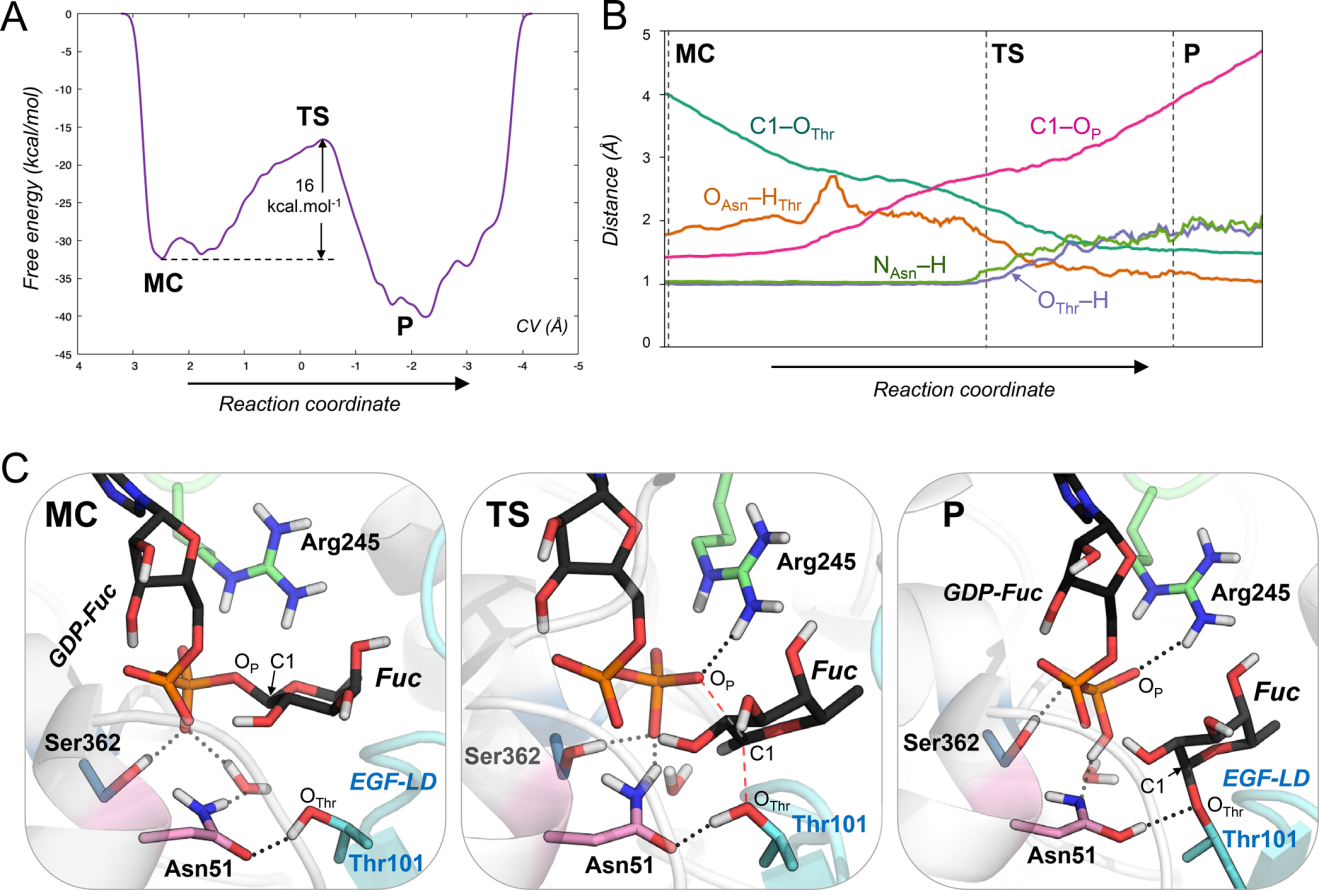
Reaction mechanism of POFUT1 obtained from QM/MM metadynamics simulations. (A) Reaction free energy profile. (B) Evolution of relevant distances along the reaction coordinate. (C) Representative structures of the Michaelis complex (MC), the reaction transition state (TS), and the reaction products (P). Hydrogen bonds are represented by dotted lines, whereas bonds being broken or formed are represented by red dashed lines. Hydrogen atoms attached to carbon atoms have been omitted for clarity.

To uncover the POFUT1 reaction mechanism, we took a snapshot of the MD-equilibrated structure in which Thr101 is in a plausible orientation for catalysis (O_Thr101_···C1 ≈ 4 Å), and we initiated QM/MM metadynamics simulations of the glycosyltransfer reaction. The QM region (Figure S5) was chosen to include part of the GDP-Fuc (the two phosphates and the fucose moiety), the side chain of Asn51, and the active site water molecule (55 QM atoms, 75 170 MM atoms). One collective variable (CV) was used to drive the reaction from reactants (GDP-Fuc + EGF-LD) to products (GDP + fucosylated EGF-LD). The CV was selected as involving the two main covalent bonds that need to be broken and formed, respectively, during the chemical reaction: the distance between the fucose anomeric carbon and the GDP phosphate (C1–O_P_, the glycosidic bond to be broken, Figure 1B) and the distance between the hydroxyl oxygen atom of Thr101 and the fucose anomeric carbon (C1–O_Thr_, the glycosidic bond to be formed). This choice of CV does not self-select who deprotonates Thr101, thus any of the mechanisms that had been previously proposed, namely, the direct deprotonation of Thr101 by the β-phosphate of GDP or the indirect deprotonation mediated by a water molecule, are possible. The chosen CV does not preclude a mechanism via Asn51 either.

The QM/MM metadynamics simulation successfully drove the reactants (GDP-Fuc + EGF-LD) toward the products (GDP + fucosylated EGF-LD). The free energy profile reconstructed from the simulation, shown in Figure 3A, shows that the reaction is exergonic. The computed energy barrier, 16 kcal mol^–1^, is in good agreement with the values estimated from experimental reaction rates (17–19 kcal mol^–1^),^16,30,31^ which gives confidence in our model. Interestingly, the reaction free energy profile features a single transition state, indicative of a concerted reaction. Additional QM/MM MD simulations of the commitment probability in the region around the TS (see SI) did not show evidence of any even short-lived intermediate. Therefore, our simulations are consistent with an S_N_2 reaction for POFUT1, as reported for other inverting GTs^3,18,26^ and contrary to the S_N_1 type of reaction that was previously suggested in structural studies.^15,16^

Analysis of the species along the reaction coordinate (Figure 3B,C) revealed the details of the chemical reaction mechanism. At the Michaelis complex (MC), the acceptor threonine forms a hydrogen bond with the amide oxygen of Asn51. Such a hydrogen bond was previously observed in the crystal structure of POFUT1 in complex with GDP and the EGF-LD acceptor.^16^ The reaction begins by the approach of the acceptor threonine to the donor anomeric carbon. The GDP-fucose glycosidic bond becomes partially broken at the transition state (TS; C1–O_P_ = 2.56 Å, Table S1), in which the new bond between the Thr oxygen and the C1 atom is partially formed (C1–O_Thr_ = 2.18 Å; Table S1). At the same time, Arg245 tightens its interaction with O_p_ (O_P_··H_Arg245_ decreases from 2.30 Å at the MC to 1.79 Å at the TS, Table S1), assisting the cleavage of the glycosidic bond. The fucosyl ring changes conformation during the reaction. It evolves from an inverted chair (^1^*C*_4_) at MC, which is the most stable conformation of L-fucose,^32^ to a distorted half-chair conformation (^3^*H*_4_) at the TS, consistent with the formation of an oxocarbenium-ion like species.

Significant atomic rearrangements in the active site take place as the Thr101 hydroxyl group approaches the C1 atom of GDP-Fuc. As shown in Figure 3C, the Asn51 side chain slightly rotates such that its amide nitrogen interacts with the β-phosphate. At the TS, the Asn51 forms tight hydrogen bonds with both Thr101 and the β-phosphate (Figure 3C). Eventually, the amide carbonyl group of Asn51 abstracts the Thr101 proton, and simultaneously, its amide amino group delivers a proton to the β-phosphate (as shown in Figure 3B, both N_Asn_–H and O_Thr_–H distances evolve together), resulting in Asn51 in the imidic acid tautomeric form. While the Thr101 and Asn51 protons are being transferred, the new glycosidic bond forms and the fucose returns to its most stable ^1^*C*_4_ conformation (P in Figure 3C). Therefore, the simulations reveal that the β-phosphate is the ultimate base that accepts the proton of the incoming Thr nucleophile, with Asn51 mediating proton transfer (Figure 1C).

The involvement of the active site Asn in the chemical reaction might seem surprising a priori, since it does not agree with the main mechanisms that were previously put forward (Figure 2B). Nevertheless, it shares some similarities with them. For instance, the final recipient of the Thr101 proton is an oxygen atom of the β-phosphate, as previously proposed. In the computed reaction pathway, the proton does not “arrive” to GDP directly from Thr101—or indirectly via a bridging water molecule—as previously assumed but via an active site residue (Asn51) that acts as a proton shuttle, as hypothesized by Li et al.^16^ The fact that Asn51 was not included in the collective variable used in the simulation reinforces this result, as the system just took the most favorable reaction pathway. A slight rotational motion of Asn51 places it such as to bridge Thr101 with the GDP β-phosphate in an optimal configuration for catalysis (Figure 3C). Additional simulations avoiding proton transfer toward Asn51 (SI page S4) did not lead to the direct proton transfer mechanism of Figure 2B, which is not surprising as a water molecule does not optimally connect Thr101 with the GDP β-phosphate.

To investigate further the role of the active site Asn in catalysis, we performed kinetic experiments on a *Ce*POFUT1 variant in which the corresponding residue (Asn43) is replaced by either an Ala or an Asp. To validate that both mutants were stable and properly folded, we estimated their melting temperatures (*T*_m_) by performing a thermal shift-assay experiment. *T*_m_ values for Asn43Ala and Asn43Asp mutants, including those for the *Ce*POFUT1 wild type, were highly similar and ∼50 °C (Figure S7 and Table S2), implying that the mutations did not alter the stability and likely the folding of *Ce*POFUT1.

As expected from the poor activity of the Asn43Ala mutant in the GDP-Fuc hydrolysis experiment,^15^ this mutant was completely inactive for glycosyltransfer (Figure 4A). Even though this does not prove a catalytic role for the active site Asn, it is consistent with it. Concerning the Asn43Asp mutant, we first note that replacement of Asn by Asp was expected to restore enzyme activity, since Asp is one of the residues typically present in inverting GTs, including POFUT2.^33^ However, Asn43Asp *Ce*POFUT1 showed an unexpected complete loss of activity (Figure 4A). Additional MD simulations on Asn51Asp *Mm*POFUT1 explained this result. The simulations showed that the side chain of Asp51 rotates away from the active site with respect to wild type *Mm*POFUT1 (Figure 4B), most likely due to repulsive electrostatic interactions with the GDP β-phosphate, while water molecules penetrate in the active site. This scenario is not very different from the one observed for the Ala variant, which is also inactive. In this configuration, Asp51 cannot act as catalytic base, and thus the enzyme cannot work.

**Figure 4:**
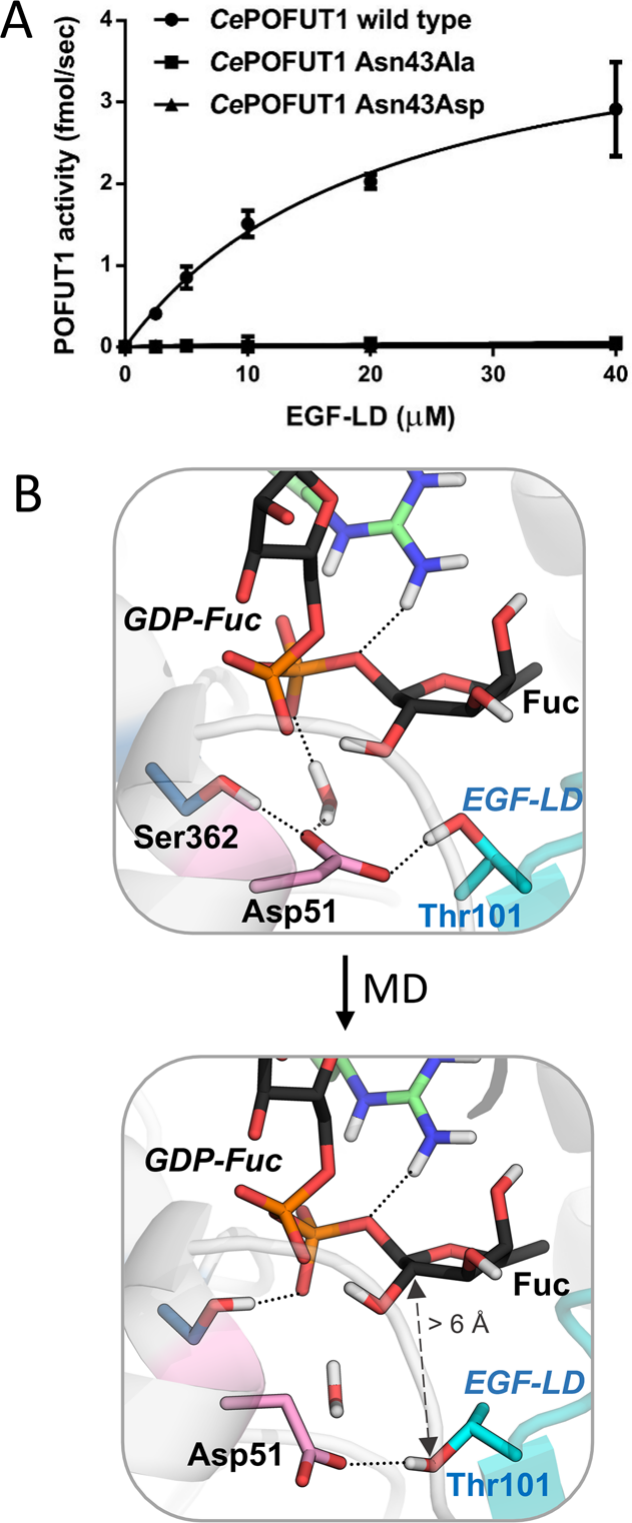
Effect of the Asn43/Asn51 mutants. (A) Kinetic results comparing the wild type *Ce*POFUT1 with the Asn43Ala and Asn43Asp variants. Acceptor (human factor IX EGF-LD)-dependent enzymatic activities of the recombinant CePOFUT1 proteins are shown. Values indicate mean ± SD. Assays were performed for 10 min at 37 °C, pH 7.0, with 10 μM GDP-fucose (details in SI). For the WT enzyme, the apparent *k*_cat_ is 0.2 s^–1^, and the apparent *K*_m_ for EGF-LD is 21 μM. (B) Evolution of the *Mm*POFUT1 Asn51Asp mutant active site during MD simulations.

The presence of an asparagine residue as an acid/base catalyst in GTs is unusual. Inverting GTs typically involve an aspartate, a glutamate, or even a histidine as a catalytic base.^18^ However, Asn is known to act as an acid/base residue in enzymes that catalyze light-activated processes.^34^ Amide–imide tautomerization has also been proposed, although not confirmed, for glycoprocessing enzymes such as families 45 and 85 glycosidases.^35,36^ The computed mechanism for *Mm*POFUT1 explains why the active site Asn, conserved among species, is an essential residue. Once the reaction takes place, its most stable amide form can be easily recovered as water molecules enter the active site (Δ*G*^‡^ for imide-amide tautomerization in water is ∼8 kcal/mol, and it requires only one water molecule).^37^

An interesting question that emerges from our study is, why has nature placed a catalytic Asn in a GT active site rather than Asp or Glu? We think that this is ultimately dictated by the acceptor specificity of POFUT1, which can only fucosylate certain types of peptide domains. The particular structural arrangement of the donor and acceptor in the POFUT1 active site, with the acceptor nucleophilic hydroxyl group being close to the donor β-phosphate and oriented toward it, unlike in classical inverting GTs,^25^ precludes the presence of a canonical general base in the active site.

In summary, QM/MM metadynamics simulations have revealed for the first time the reaction mechanism by which POFUT1 glycosylates its natural substrate EGF-LD Factor VII or other EGF-like substrates from GDP-Fuc. The chemical reaction follows an S_N_2 mechanism, as in classical inverting glycosyltransferases, but an asparagine residue rather than a basic residue deprotonates the hydroxyl group of the Thr nucleophile. This is possible in POFUT1 because Asn51 can reorient such that it bridges the nucleophile residue with the negatively charged β-phosphate, shuttling a proton between the two, a scenario that is reminiscent to the one predicted, but not yet confirmed, for other carbohydrate-active enzymes.^35,36^ This crucial role of Asn51 explains why mutation of this residue to either Ala or Asp results in a lack of enzyme activity. Our data solve the conundrum of how the enzyme works in the absence of a canonical basic residue in the active site that assists catalysis and offer a plausible mechanism for other GTs that might contain an Asn residue in a similar position to that of POFUT1. The mechanistic insight provided by our study will also be important for the development of mechanism-based inhibitors of POFUT1 to target diseases associated with Notch signal transduction.

## Supporting Information Available

Computational details and experimental methods (PDF)

## References

[ref1] Schjoldager K. T.; Narimatsu Y.; Joshi H. J.; Clausen H. Global View of Human Protein Glycosylation Pathways and Functions. Nat. Rev. Mol. Cell Biol. 2020, 21, 729–749. 10.1038/s41580-020-00294-x. 33087899

[ref2] Varki A. Biological Roles of Glycans. Glycobiology 2017, 27, 3–49. 10.1093/glycob/cww086. 27558841 PMC5884436

[ref3] Lairson L. L.; Henrissat B.; Davies G. J.; Withers S. G. Glycosyltransferases: Structures, Functions, and Mechanisms. Annu. Rev. Biochem. 2008, 77, 521–55. 10.1146/annurev.biochem.76.061005.092322. 18518825

[ref4] Moremen K. W.; Haltiwanger R. S. Emerging Structural Insights Into Glycosyltransferase-Mediated Synthesis of Glycans. Nat. Chem. Biol. 2019, 15, 853–864. 10.1038/s41589-019-0350-2. 31427814 PMC6820136

[ref5] Andre I.; Potocki-Veronese G.; Barbe S.; Moulis C.; Remaud-Simeon M. CAZyme Discovery and Design for Sweet Dreams. Curr. Opin. Chem. Biol. 2014, 19, 17–24. 10.1016/j.cbpa.2013.11.014. 24780275

[ref6] Schneider M.; Al-Shareffi E.; Haltiwanger R. S. Biological Functions of Fucose in Mammals. Glycobiology 2017, 27, 601–618. 10.1093/glycob/cwx034. 28430973 PMC5458543

[ref7] Holdener B. C.; Haltiwanger R. S. Protein O-fucosylation: Structure and Function. Curr. Opin. Struct. Biol. 2019, 56, 78–86. 10.1016/j.sbi.2018.12.005. 30690220 PMC6656592

[ref8] Bray S. J. Notch Signalling: a Simple Pathway Becomes Complex. Nat. Rev. Mol. Cell Biol. 2006, 7, 678–89. 10.1038/nrm2009. 16921404

[ref9] Okajima T.; Xu A.; Lei L.; Irvine K. D. Chaperone Activity of Protein O-fucosyltransferase 1 Promotes Notch Receptor Folding. Science 2005, 307, 1599 10.1126/science.1108995. 15692013

[ref10] McMillan B. J.; Zimmerman B.; Egan E. D.; Lofgren M.; Xu X.; Hesser A.; Blacklow S. C. Structure of Human POFUT1, its Requirement in Ligand-Independent Oncogenic Notch Signaling, and Functional Effects of Dowling-Degos Mutations. Glycobiology 2017, 27, 777–786. 10.1093/glycob/cwx020. 28334865 PMC5881682

[ref11] Jundt F.; Schwarzer R.; Dorken B. Notch Signaling in Leukemias and Lymphomas. Curr. Mol. Med. 2008, 8, 51–9. 10.2174/156652408783565540. 18289013

[ref12] Du Y.; Li D.; Li N.; Su C.; Yang C.; Lin C.; Chen M.; Wu R.; Li X.; Hu G. POFUT1 Promotes Colorectal Cancer Development Through the Activation of Notch1 Signaling. Cell Death Dis. 2018, 9, 995 10.1038/s41419-018-1055-2. 30250219 PMC6155199

[ref13] Okajima T.; Irvine K. D. Regulation of Notch Signaling by O-Linked Fucose. Cell 2002, 111, 893–904. 10.1016/S0092-8674(02)01114-5. 12526814

[ref14] Shi S.; Stanley P. Protein O-Fucosyltransferase 1 is an Essential Component of Notch Signaling Pathways. Proc. Natl. Acad. Sci. U. S. A. 2003, 100, 5234–9. 10.1073/pnas.0831126100. 12697902 PMC154328

[ref15] Lira-Navarrete E.; Valero-Gonzalez J.; Villanueva R.; Martinez-Julvez M.; Tejero T.; Merino P.; Panjikar S.; Hurtado-Guerrero R. Structural Insights into the Mechanism of Protein *O-*Fucosylation. PLoS One 2011, 6, e25365 10.1371/journal.pone.0025365. 21966509 PMC3180450

[ref16] Li Z.; Han K.; Pak J. E.; Satkunarajah M.; Zhou D.; Rini J. M. Recognition of EGF-like Domains by the Notch-Modifying *O-*Fucosyltransferase POFUT1. Nat. Chem. Biol. 2017, 13, 757–763. 10.1038/nchembio.2381. 28530709

[ref17] Lira-Navarrete E.; Hurtado-Guerrero R. A Perspective on Structural and Mechanistic Aspects of Protein *O-*Fucosylation. Acta Crystallogr., Sect. F: Struct. Biol. Commun. 2018, 74, 443–450. 10.1107/S2053230X18004788. 30084393 PMC6096484

[ref18] Teze D.; Coines J.; Fredslund F.; Dubey K. D.; Bidart G. N.; Adams P. D.; Dueber J. E.; Svensson B.; Rovira C.; Welner D. H. O-/N-/S-Specificity in Glycosyltransferase Catalysis: From Mechanistic Understanding to Engineering. ACS Catal. 2021, 11, 1810–1815. 10.1021/acscatal.0c04171.

[ref19] Laio A.; VandeVondele J.; Rothlisberger U. A Hamiltonian Electrostatic Coupling Scheme for Hybrid Car-Parrinello Molecular Dynamics Simulations. J. Chem. Phys. 2002, 116, 6941–6947. 10.1063/1.1462041.

[ref20] Laio A.; Parrinello M. Escaping Free-Energy Minima. Proc. Natl. Acad. Sci. U. S. A. 2002, 99, 12562–12566. 10.1073/pnas.202427399. 12271136 PMC130499

[ref21] Raich L.; Nin-Hill A.; Ardevol A.; Rovira C. Enzymatic Cleavage of Glycosidic Bonds: Strategies on How to Set Up and Control a QM/MM Metadynamics Simulation. Methods Enzymol. 2016, 577, 159–83. 10.1016/bs.mie.2016.05.015. 27498638

[ref22] Raich L.; Borodkin V.; Fang W.; Castro-Lopez J.; van Aalten D. M.; Hurtado-Guerrero R.; Rovira C. A Trapped Covalent Intermediate of a Glycoside Hydrolase on the Pathway to Transglycosylation. Insights from Experiments and Quantum Mechanics/Molecular Mechanics Simulations. J. Am. Chem. Soc. 2016, 138, 3325–32. 10.1021/jacs.5b10092. 26859322

[ref23] Coines J.; Raich L.; Rovira C. Modeling Catalytic Reaction Mechanisms in Glycoside Hydrolases. Curr. Opin. Chem. Biol. 2019, 53, 183–191. 10.1016/j.cbpa.2019.09.007. 31731209

[ref24] Nin-Hill A.; Rovira C. The Catalytic Reaction Mechanism of the β-Galactocerebrosidase Enzyme Deficient in Krabbe Disease. ACS Catal. 2020, 10, 12091–12097. 10.1021/acscatal.0c02609.

[ref25] Morais M. A. B.; Coines J.; Domingues M. N.; Pirolla R. A. S.; Tonoli C. C. C.; Santos C. R.; Correa J. B. L.; Gozzo F. C.; Rovira C.; Murakami M. T. Two Distinct Catalytic Pathways for GH43 Xylanolytic Enzymes Unveiled by X-ray and QM/MM Simulations. Nat. Commun. 2021, 12, 367 10.1038/s41467-020-20620-3. 33446650 PMC7809346

[ref26] Darby J. F.; Gilio A. K.; Piniello B.; Roth C.; Blagova E.; Hubbard R. E.; Rovira C.; Davies G. J.; Wu L. Substrate Engagement and Catalytic Mechanisms of N-Acetylglucosaminyltransferase V. ACS Catal. 2020, 10, 8590–8596. 10.1021/acscatal.0c02222.

[ref27] Ardèvol A.; Rovira C. The Molecular Mechanism of Enzymatic Glycosyl Transfer with Retention of Configuration: Evidence for a Short-Lived Oxocarbenium-like Species. Angew. Chem., Int. Ed. 2011, 50, 10897–901. 10.1002/anie.201104623. 21953735

[ref28] Lira-Navarrete E.; Iglesias-Fernández J.; Zandberg W. F.; Companon I.; Kong Y.; Corzana F.; Pinto B. M.; Clausen H.; Peregrina J. M.; Vocadlo D. J.; Rovira C.; Hurtado-Guerrero R. Substrate-Guided Front-Face Reaction Revealed by Combined Structural Snapshots and Metadynamics for the Polypeptide N-Acetylgalactosaminyltransferase 2. Angew. Chem., Int. Ed. 2014, 53, 8206–8210. 10.1002/anie.201402781. 24954443

[ref29] Bilyard M. K.; Bailey H. J.; Raich L.; Gafitescu M. A.; Machida T.; Iglesias-Fernandez J.; Lee S. S.; Spicer C. D.; Rovira C.; Yue W. W.; Davis B. G. Palladium-Mediated Enzyme Activation Suggests Multiphase Initiation of Glycogenesis. Nature 2018, 563, 235–240. 10.1038/s41586-018-0644-7. 30356213

[ref30] Wang Y.; Spellman M. W. Purification and Characterization of a GDP-fucose:Polypeptide Fucosyltransferase from Chinese Hamster Ovary Cells. J. Biol. Chem. 1998, 273, 8112–8. 10.1074/jbc.273.14.8112. 9525914

[ref31] Wang Y.; Shao L.; Shi S.; Harris R. J.; Spellman M. W.; Stanley P.; Haltiwanger R. S. Modification of epidermal growth factor-like repeats with O-fucose. Molecular cloning and expression of a novel GDP-fucose protein O-fucosyltransferase. J. Biol. Chem. 2001, 276, 40338–45. 10.1074/jbc.M107849200. 11524432

[ref32] Lammerts van Bueren A.; Fayers-Kerr J.; Luo B.; Zhang Y.; Sollogoub M.; Bleriot Y.; Rovira C.; Davies G. J Analysis of the Reaction Coordinate of α-L-Fucosidases: a Combined Structural and Quantum Mechanical Approach. J. Am. Chem. Soc. 2010, 132, 1804 10.1021/ja908908q. 20092273

[ref33] Valero-Gonzalez J.; Leonhard-Melief C.; Lira-Navarrete E.; Jimenez-Oses G.; Hernandez-Ruiz C.; Pallares M. C.; Yruela I.; Vasudevan D.; Lostao A.; Corzana F.; Takeuchi H.; Haltiwanger R. S.; Hurtado-Guerrero R. A Proactive role of Water Molecules in Acceptor Recognition by Protein O-fucosyltransferase 2. Nat. Chem. Biol. 2016, 12, 240–6. 10.1038/nchembio.2019. 26854667 PMC4845761

[ref34] Grigorenko B. L.; Khrenova M. G.; Nemukhin A. V. Amide-Imide Tautomerization in the Glutamine Side Chain in Enzymatic and Photochemical Reactions in Proteins. Phys. Chem. Chem. Phys. 2018, 20, 23827–23836. 10.1039/C8CP04817G. 30202846

[ref35] Abbott D. W.; Macauley M. S.; Vocadlo D. J.; Boraston A. B. *Streptococcus Pneumoniae* Endohexosaminidase D, Structural and Mechanistic Insight into Substrate-Assisted Catalysis in Family 85 Glycoside Hydrolases. J. Biol. Chem. 2009, 284, 11676–89. 10.1074/jbc.M809663200. 19181667 PMC2670171

[ref36] Nakamura A.; Ishida T.; Kusaka K.; Yamada T.; Fushinobu S.; Tanaka I.; Kaneko S.; Ohta K.; Tanaka H.; Inaka K.; Higuchi Y.; Niimura N.; Samejima M.; Igarashi K. “Newton’s Cradle” Proton Relay with Amide-Imidic Acid Tautomerization in Inverting Cellulase Visualized by Neutron Crystallography. Sci. Adv. 2015, 1, e1500263 10.1126/sciadv.1500263. 26601228 PMC4643802

[ref37] Constantino E.; Solans-Monfort X.; Sodupe M.; Bertran J. Basic and Acidic Bifunctional Catalysis: Application to the Tautomeric Equilibrium of Formamide. Chem. Phys. 2003, 295, 151–158. 10.1016/j.chemphys.2003.08.003.

