# Supplementary material for: Asparagine Tautomerization in Glycosyltransferase Catalysis. The Molecular Mechanism of Protein O-Fucosyltransferase 1
Source: ACS Catal. 2021 Jul 23;11(15):9926–32. doi: 10.1021/acscatal.1c01785 (PMC8631701; doi:10.1021/acscatal.1c01785)
Supplement: Supplementary file 1 — cs1c01785_si_001.pdf [file cs1c01785_si_001.pdf]

SUPPORTING INFORMATION

Asparagine tautomerization in glycosyltransferase catalysis. The molecular mechanism of protein *O*-fucosyltransferase 1

Beatriz Piniello[1], Erandi Lira-Navarrete[2], Hideyuki Takeuchi[3,†], Megumi Takeuchi[3], Robert S. Hal-tiwanger[3], Ramón Hurtado-Guerrero[2,4,5,*] and Carme Rovira[1,6,*]

[1]Departament de Química Inorgànica i Orgànica (Secció de Química Orgànica) and Institut de Química Teòrica i Computacional (IQTCUB), Universitat de Barcelona, Martí i Franquès 1, 08028 Barcelona, Spain. [2]Institute of Biocomputation and Physics of Complex Systems (BIFI), University of Zaragoza, Mariano Esquillor s/n, Campus Rio Ebro, Edificio I+D, 50018 Zaragoza, Spain. [3]Department of Biochemistry and Molecular Biology, Complex Carbohydrate Research Center, The University of Georgia, Athens, GA 30602. [4]Fundación ARAID, 50018 Zaragoza, Spain. [5]Copenhagen Center for Glycomics, Department of Cellular and Molecular Medicine, University of Copenhagen, 1017 Copenhagen, Denmark. [6]Institució Catalana de Recerca i Estudis Avançats, Passeig Lluís Companys 23, 08010 Barcelona, Spain.

Corresponding Authors:

*Ramón Hurtado-Guerrero (rhurtado@bifi.es)
*Carme Rovira (c.rovira@ub.edu)

Contents

1. Computational details

1.1. Model building

1.2. Classical MD simulations

1.3. QM/MM MD simulations

1.4. QM/MM metadynamics simulations

2. Experimental methods

2.1. Site-directed mutagenesis

2.2. Purification of wild type CePOFUT1 and the Asn43Ala and Asn43Asp mutants

2.3. Enzyme kinetics

2.4. Thermal shift analysis

3. Tables and Figures

4. Cartesian coordinates of states R, TS and P

5. References

1. Computational details

1.1. Model building

The starting model structure for the simulations was build from the available crystal structures. A ternary complex of wild type POFUT1 with intact donor (GDP-Fuc) and acceptor protein (EGF-LD) is not available. However, there are two high resolution structures[1] that can be used to build such a complete ternary complex. On one hand, there is a structure of *Mm*POFUT1 in complex with GDP-Fuc and a mutant of the EGF-LD acceptor (the nucleophile Thr101 was replaced by Ala) (PDB 5KY3, at 1.53 Å resolution). On another hand, there is a structure of *Mm*POFUT1 in complex with GDP and the intact EGF-LD Factor VII acceptor (PDB 5KXH, at 1.33 Å resolution). We used the former structure as a template and reverted the Thr101Ala mutation by taking the Thr101 coordinates from the latter structure. The structure obtained is shown in Figure S2.

1.2. Classical MD simulations

The protonation states of the charged amino acid residues (Asp, Glu and His) were taken according to protein environment and predictions made by the MolProbity and propKa softwares considering pH 8.5.[2-3] A total number of 23001 water molecules were present within a radius of 15 Å from the protein and a Na^+ ion was added to neutralize the charge of the system. The simulation box dimensions were 81.384 x 102.753 x 90.406 $Å^3$. Molecular dynamics (MD) simulations were performed using Amber18 software.[4] The protein was modeled using the FF14SB force field.[5] The carbohydrate substrate, water molecules and GDP were described with the GLYCAM06,TIP3P and GAFF force fields,[6-7] respectively. The MD simulation was carried out in several steps. First, the system was minimized, holding the protein and substrate fixed, followed by energy minimization on the entire system. To gradually reach the desired temperature, weak spatial constraints were initially added to the protein and substrate, while water molecules and the sodium ion were allowed to move freely at 100 K. The constraints were then removed and the working temperature of 300K was reached after two more 100 K heating steps in the NVT ensemble. Afterwards, the density was converged up to water density at 300 K in the NPT ensemble and the simulation was extended to 100 ns in the NPT ensemble. Three MD replicas were considered to increase the sampling of the configurational space.

1.3. QM/MM MD simulations

The QM/MM simulations were started from a suitable frame extracted from the equilibrated classical MD trajectory. The simulations were performed using the method developed by Laio et al.,[8] which combines Car-Parrinello MD,[9] based on DFT, with force-field MD.[10] The electrostatic interactions between the QM and MM regions were handled by a fully Hamiltonian coupling scheme,[8] where the short-range electrostatic interactions between the QM and the MM regions are explicitly taken into account for all atoms. Kohn-Sham orbitals were expanded in a plane wave basis set with a kinetic energy cutoff of 70 Ry. Troullier-Martins *ab initio* pseudopotentials[11] were used for all elements. The PBE functional[12] in the generalized gradient-corrected approximation of DFT was used, in consistency with previous works on glycosyltransferase reaction mechanisms.[13-15] A constant temperature of 300 K was reached by coupling the system to a Nosé-Hoover thermostat.[16] A time step of 0.12 fs and a fictitious electron mass of 700 a.u. for the Car-Parrinello Lagrangian[9] were used. A total of 55 QM atoms were employed, including the fucose moiety, the two phosphate groups of GDP, the side chains of Thr101 and Asn51 and a water molecule, as well as 75170 MM atoms. The QM/MM interface was modeled by the use of capping hydrogen atoms that saturate the QM region, which was enclosed in an isolated supercell of size 17.62 x 16.92 x 16.01 $Å^3$.

1.4. QM/MM metadynamics simulations

QM/MM metadynamics[17-19] simulations were performed to model the molecular mechanism and free energy profile of the glycosyltransfer reaction. QM/MM MD simulations were coupled with the metadynamics algorithm provided by the Plumed 2 plugin.[20] The system was re-equilibrated by QM/MM MD for 6 ps before the metadynamics algorithm was switched on. One collective variable (CV), taken as the difference of C1-O_{Thr101} and C1-O_P distances, was considered, using a hill height of 1.0 kcal/mol. The Gaussian width and deposition time were set at 0.1 Å and 30 fs (250 MD steps), respectively, according to the oscillations of the CV in an unbiased dynamics. The simulations were stopped when recrossing over the TS occurred at least once, following literature recommendations.[21] The Gaussian height was greatly diminished (0.1 kcal/mol) upon crossing/recrossing along the TS, for a better convergence and accuracy. The energy profile was completed after 884

deposited Gaussians. The computed free energy barrier was compared to the one derived from the experimentally determined catalytic rate using TS theory.[22] Structure analysis was done with VMD[23], cpptraj from the AmberTools suite, and in-house python3 programs. The computed free energy barrier was compared to the one derived from the experimentally determined catalytic rate using transition state theory (TST) and the Eyring–Polanyi expression:

$$k = \frac{k_B T}{h} \exp(-\frac{\Delta G^{\ddagger}}{RT})$$

Where k is the experimental rate constant, k_B, h and R are the Bolzmann, Planck and gas constants, respectively. The formula assumes that the TST transmission coefficient of the is 1, i.e. TS recrossing, tunneling and nonequilibrium contributions are neglected.[22] According to the above formula, a rate constant of 0.2 s^{-1} (data for *Ce*POFUT1 obtained in this work) corresponds to an energy barrier of 19 kcal/mol. Previously reported kinetic data for *Mm*POFUT1, Chinese hamster and human POFUT1 correspond to free energy values in the range 17-19 kcal/mol.[1, 24-25] The reaction TS was further validated from commitment analyses. Briefly, a collection of points around the TS of the free energy profile were defined. A series of QM/MM MD simulations with random initial velocities were performed at each point and the probability of collapsing either to reactants (MC) or products (P) was evaluated. A point in which 50% probability was identified, which turned out to be very close ($C1\text{-}O_{Thr101}$ = 2.18 Å, $C1\text{-}O_{P}$ = 2.56 Å) to the one obtained from analysis of the reaction free energy profile ($C1\text{-}O_{Thr101}$ = 2.28 ± -0.11 Å, $C1\text{-}O_{P}$ = 2.69 ± 0.10 Å).

To check whether there could be an alternative mechanism that does not involve Asn51, a metadynamics simulation was designed in which Asn51 was not part of the QM region, and thus cannot react. The results obtained showed that the reaction can still take place via transfer of the Thr101 proton to the β-phosphate via the fucose 2-OH group. However, the free energy barrier of this process was much higher than the one previously obtained. This indicates that the only plausible reaction mechanism is the one involving Asn51 tautomerization.

2. Experimental methods

2.1. Site-directed mutagenesis

The *Ce*POFUT1 Asn43Asp mutant was generated following a standard site-directed mutagenesis protocol by GenScript (quick change), and using the vector pPICZαA*cepofut1* (T26-A382).[26]

2.2. Purification of wild type CePOFUT1 and the Asn43Ala and Asn43Asp mutants

The wild type *Ce*POFUT1 and the Asn43Ala and Asn43Asp mutants were purified as previously described for the wild type enzyme.[26]

2.3. Enzyme kinetics

As most GT-B glycosyltransferases, POFUT1 does not require a metal for activity. However, and depending on the species, Mn^{+2} might have a stabilizing effect on the Chinese hamster ovary POFUT1 by increasing its glycosyltransfer activity, or a slightly negative effect on the *Ce*POFUT1 GDP-Fuc hydrolysis experiment.[24, 26]

The concentrations of *Ce*POFUT1 proteins were quantitated by 10% SDS-PAGE followed by Coomassie staining using bovine serum albumin as standards. POFUT1 enzyme assays were performed as previously described.[27] Briefly, a 10-μl reaction mixture contained 50 mM HEPES pH 7.0, 10 mM $MnCl_2$, the indicated concentrations of the bacterially expressed EGF-LD from human factor IX, 0.3 μM GDP-[^{3}H]fucose, 10 μM GDP-fucose, 0.5% Nonidet-P40, and 1 ng of purified *Ce*POFUT1 proteins. The reaction was performed at 37ºC for 10 min and stopped by adding 900 μl of 100 mM EDTA pH 8.0. The sample was loaded onto a C18 cartridge (100 mg, Agilent). After the cartridge was washed with 5 ml of H_2O, the bound EGF-LDs were eluted with 1 ml of 80% methanol. Incorporation of [^{3}H]fucose into the EGF-LDs was determined by scintillation counting of the eluates. Reactions without acceptor EGF-LDs were performed as a background control. Triplicate assays were performed for each data point and standard deviations were determined.

2.4. Thermal shift analysis

Analysis of the protein stability of *Ce*PoFUT1 wild type, the Asn43Ala mutant and the Asn43Asp mutant was determined through a thermal shift analysis. 5 µM of CePOFUT1 wild

type, the Asn43Ala mutant and the Asn43Asp mutant in 20 mM HEPES pH=7.5, 150 mM NaCl, were incubated with 5x concentrated SYPRO™ orange dye (ThermoFisher Scientific) in a final volume of 100 µl. SYPRO™ orange dye emission was monitored at different temperatures starting at 25ºC and ending at 100ºC with increments of 1ºC.[28] The assay was performed in a Stratagene Mx3005P qPCR real-time instrument (Agilent Technologies), using excitation and emission filters of 492 and 610 nm, respectively. Each experiment was performed in triplicate.

Data analysis were performed with GraphPad PRISM software. To obtain T_m, a Boltzmann model was used to fit the fluorescence imaging data:

$$I = (A+B ((B-A)/(1+\exp (T_m-T)/C))$$

where *I* is the fluorescence intensity at temperature *T*, *A* and *B* are pre-transitional and post-transitional fluorescence intensities, respectively, and *C* is a slope factor.

3. Tables and Figures

Table S1. Main structural parameters in the active site along the reaction coordinate.

**Distance**[a]	**MC**	**TS**[b]	**P**
C1 ⋯ O_{Thr101}	3.90 ± 0.05	2.18	1.53 ± 0.13
C1 – O_P	1.43 ± 0.04	2.56	3.79 ± 0.14
C1 – O5	1.44 ± 0.03	1.33	1.39 ± 0.04
$(C – N)_{Asn51}$	1.35 ± 0.02	1.34	1.30 ± 0.04
$(O – H)_{Thr101}$	1.00 ± 0.03	1.08	1.85 ± 0.62
$(N – H)_{Asn51}$	1.03 ± 0.03	1.15	1.83 ± 0.76
O_{Asn51} – H_{Thr101}	1.83 ± 0.12	1.56	1.18 ± 0.23
O_P⋯H_{Arg245}	2.30 ± 0.24	1.79	1.86 ± 0.07

[a] The depicted connectivity refers to the MC state. [b] Values obtained from committor analysis

Table S2. T_m values of *Ce*POFUT1 wild type, the Asn43Ala mutant and the Asn43Asp mutant.

**Protein**	**T_m (ºC)**
*Ce*PoFUT1 wild type	49.61 ± 0.010
*Ce*PoFUT1 Asn43Ala	51.19 ± 0.062
*Ce*PoFUT1 Asn43Asp	53.25 ± 0.373

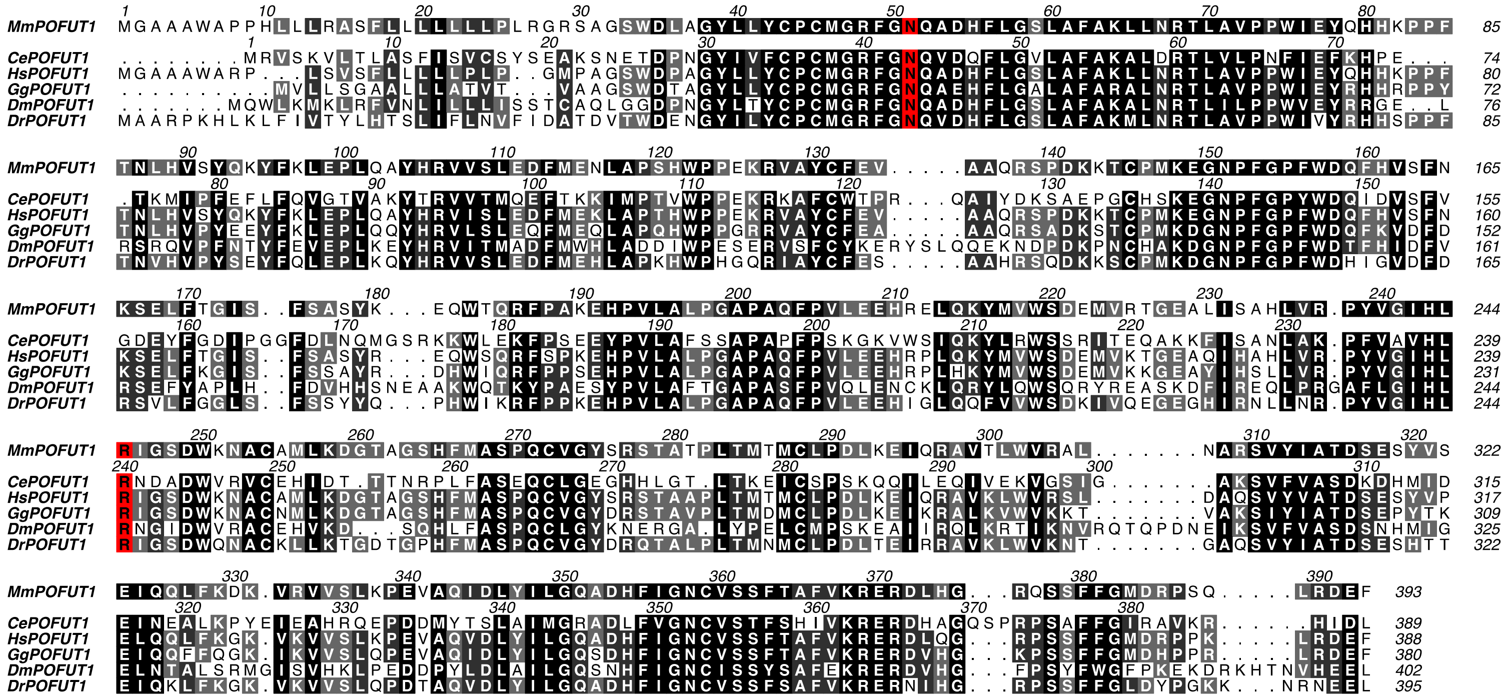

Figure S1. Multiple sequence alignment of POFUT1s. Multiple sequence alignment of the GT65 family members *Mm*POFUT1, *Ce*POFUT1, human POFUT1 (*Hs*POFUT1), *Gallus gallus* POFUT1 (*Gg*POFUT1), *Drosophila melanogaster* POFUT1 (*Dm*POFUT1) and *Danio renio* POFUT1 (*Dr*POFUT1). The conserved Asn and Arg residues are highlighted in red.

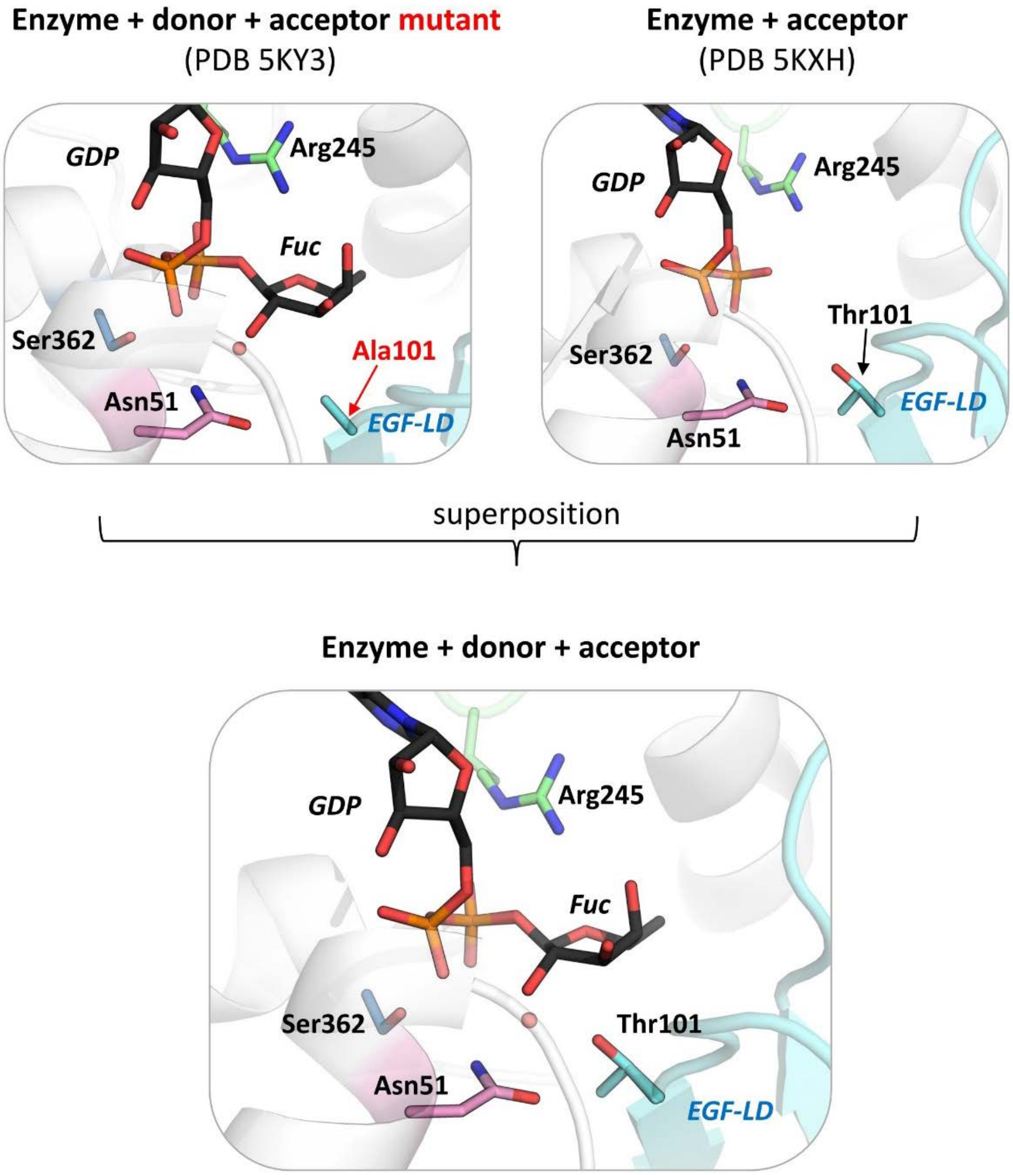

Figure S2. Ternary complex (Michaelis complex, MC) of *Mm*POFUT1 obtained from superposition of the two crystal structures of Li et al.[1]

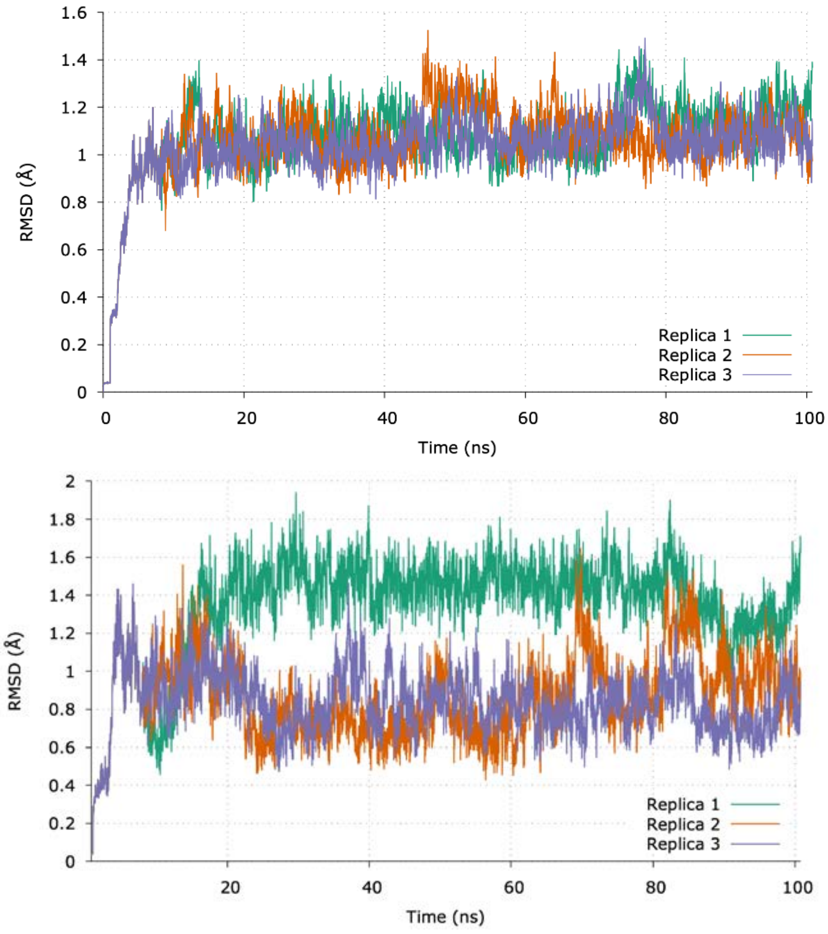

Figure S3. RMSD evolution along the classical MD simulations for the three replicas considered, for the enzyme (Top) and the EGF-LD (Bottom)

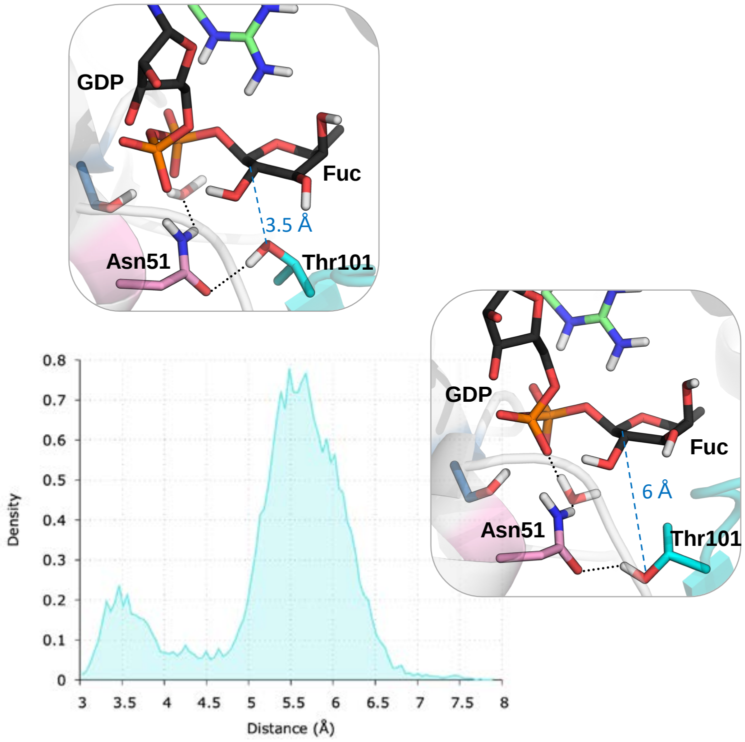

Figure S4. Rotational motion of Thr101 observed during the MD simulations.

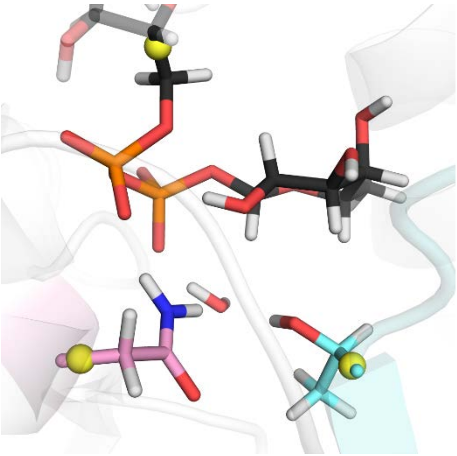

Figure S5. QM region used in the QM/MM simulations (in solid color, 55 atoms). Yellow spheres correspond to the capping hydrogen atoms.

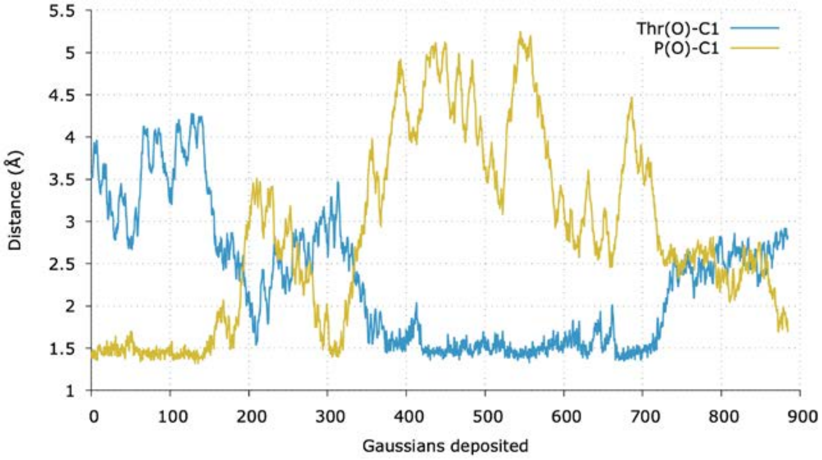

Figure S6. Evolution of the two distances included in the CV along the QM/MM metadynamics simulation of glycosyl transfer.

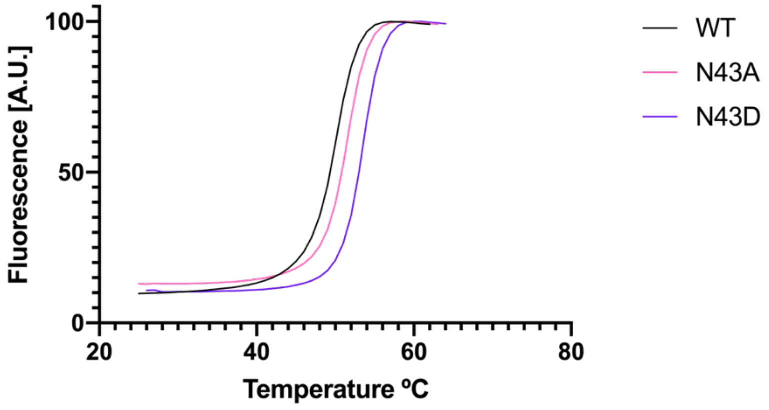

Figure S7. Thermal denaturalization curves of *Ce*POFUT1 wild type (black), the Asn43Ala mutant (magenta) and the Asn43Asp mutant (purple). Experiments were performed in triplicates. For illustration purposes, data were normalized for all samples by taking the maximum fluorescence data as 100.

4. Cartesian coordinates of states R, TS and P

The Cartesian coordinates (PDB format) of the atoms included in the QM region in states R, TS and P (representative structures shown in Figure 3C) are listed below. The TS coordinates were obtained from committor analysis, whereas those of R and P are representative snapshot at the minimum of the free energy profile.

Michaelis Complex

```
ATOM      1  CB  ASN X  21      10.114   4.974   5.296  0.00  0.00
ATOM      2  HB2 ASN X  21      10.849   5.773   5.662  0.00  0.00
ATOM      3  HB3 ASN X  21       9.986   5.097   4.212  0.00  0.00
ATOM      4  CG  ASN X  21       8.708   5.308   5.875  0.00  0.00
ATOM      5  OD1 ASN X  21       7.861   5.903   5.205  0.00  0.00
ATOM      6  ND2 ASN X  21       8.559   5.106   7.210  0.00  0.00
ATOM      7 HD21 ASN X  21       7.896   5.672   7.655  0.00  0.00
ATOM      8 HD22 ASN X  21       9.363   4.735   7.705  0.00  0.00
ATOM      9  CB  THR A 366       5.980   8.958   5.796  0.00  0.00
ATOM     10  HB  THR A 366       5.797   9.777   6.554  0.00  0.00
ATOM     11  CG2 THR A 366       4.807   8.015   5.813  0.00  0.00
ATOM     12 HG21 THR A 366       4.707   7.366   4.956  0.00  0.00
ATOM     13 HG22 THR A 366       4.844   7.477   6.738  0.00  0.00
ATOM     14 HG23 THR A 366       3.803   8.551   5.744  0.00  0.00
ATOM     15  OG1 THR A 366       7.214   8.272   6.142  0.00  0.00
ATOM     16  HG1 THR A 366       7.224   7.438   5.602  0.00  0.00
ATOM     17  O1  GFB X 392      13.736   5.702   9.693  0.00  0.00
ATOM     18  P1  GFB X 392      12.511   6.478   9.513  0.00  0.00
ATOM     19  O2  GFB X 392      11.745   6.565   8.254  0.00  0.00
ATOM     20  O3  GFB X 392      11.507   5.963  10.793  0.00  0.00
ATOM     21  P2  GFB X 392       9.974   6.373  11.161  0.00  0.00
ATOM     22  O9  GFB X 392       9.918   7.991  10.530  0.00  0.00
ATOM     23  O10 GFB X 392       8.920   5.507  10.493  0.00  0.00
ATOM     24  O11 GFB X 392       9.902   6.523  12.667  0.00  0.00
ATOM     25  O4  GFB X 392      12.933   7.979   9.826  0.00  0.00
ATOM     26  C1  GFB X 392      13.268   8.524  11.129  0.00  0.00
ATOM     27  H4  GFB X 392      12.761   9.451  11.385  0.00  0.00
ATOM     28  H8  GFB X 392      12.997   7.725  11.925  0.00  0.00
ATOM     29  C1  0fB X 393       9.094   8.634   9.516  0.00  0.00
ATOM     30  H1  0fB X 393       8.476   7.949   8.903  0.00  0.00
ATOM     31  C2  0fB X 393       9.927   9.533   8.638  0.00  0.00
ATOM     32  H2  0fB X 393      10.655  10.084   9.276  0.00  0.00
ATOM     33  C3  0fB X 393       9.093  10.644   7.914  0.00  0.00
ATOM     34  H3  0fB X 393       8.385  10.139   7.239  0.00  0.00
ATOM     35  C4  0fB X 393       8.274  11.354   8.987  0.00  0.00
ATOM     36  H4  0fB X 393       7.650  12.132   8.469  0.00  0.00
ATOM     37  C5  0fB X 393       7.419  10.417   9.777  0.00  0.00
ATOM     38  H5  0fB X 393       6.805   9.942   9.001  0.00  0.00
ATOM     39  C6  0fB X 393       6.582  10.992  10.875  0.00  0.00
ATOM     40  H61 0fB X 393       5.919  11.821  10.511  0.00  0.00
ATOM     41  H62 0fB X 393       5.773  10.250  11.088  0.00  0.00
ATOM     42  H63 0fB X 393       7.063  11.365  11.783  0.00  0.00
ATOM     43  O5  0fB X 393       8.223   9.381  10.426  0.00  0.00
```

```
ATOM     44  O4  0fB X 393       9.122  12.076   9.857  0.00  0.00
ATOM     45  H4O 0fB X 393       8.956  13.034   9.615  0.00  0.00
ATOM     46  O3  0fB X 393      10.057  11.613   7.358  0.00  0.00
ATOM     47  H3O 0fB X 393      10.331  11.361   6.429  0.00  0.00
ATOM     48  O2  0fB X 393      10.562   8.838   7.560  0.00  0.00
ATOM     49  H2O 0fB X 393      10.998   7.963   7.819  0.00  0.00
ATOM     50  O   WAT X5021       6.488   6.143   9.007  0.00  0.00
ATOM     51  H1  WAT X5021       5.628   6.551   9.307  0.00  0.00
ATOM     52  H2  WAT X5021       6.966   5.969   9.850  0.00  0.00
ATOM     53  DUM DUM X   5      14.316   8.526  11.164  0.00  0.00
ATOM     54  DUM DUM X   5       6.138   9.509   4.858  0.00  0.00
ATOM     55  DUM DUM X   5      10.578   3.997   5.644  0.00  0.00
```

Transition state

```
ATOM      1  CB  ASN X  21      10.244   5.052   5.085  0.00  0.00
ATOM      2  HB2 ASN X  21      11.108   5.779   5.042  0.00  0.00
ATOM      3  HB3 ASN X  21       9.819   4.956   4.029  0.00  0.00
ATOM      4  CG  ASN X  21       9.212   5.688   6.014  0.00  0.00
ATOM      5  OD1 ASN X  21       8.465   6.532   5.433  0.00  0.00
ATOM      6  ND2 ASN X  21       9.186   5.437   7.335  0.00  0.00
ATOM      7 HD21 ASN X  21       8.903   5.796   8.392  0.00  0.00
ATOM      8 HD22 ASN X  21      10.032   4.935   7.608  0.00  0.00
ATOM      9  CB  THR A 366       6.373   8.999   6.270  0.00  0.00
ATOM     10  HB  THR A 366       5.986   9.715   6.964  0.00  0.00
ATOM     11  CG2 THR A 366       5.598   7.762   6.360  0.00  0.00
ATOM     12 HG21 THR A 366       6.100   6.998   5.837  0.00  0.00
ATOM     13 HG22 THR A 366       5.669   7.455   7.410  0.00  0.00
ATOM     14 HG23 THR A 366       4.525   7.766   6.134  0.00  0.00
ATOM     15  OG1 THR A 366       7.744   8.777   6.581  0.00  0.00
ATOM     16  HG1 THR A 366       8.035   7.801   6.235  0.00  0.00
ATOM     17  O1  GFB X 392      13.857   5.822   9.271  0.00  0.00
ATOM     18  P1  GFB X 392      12.485   6.533   9.213  0.00  0.00
ATOM     19  O2  GFB X 392      11.877   6.803   7.831  0.00  0.00
ATOM     20  O3  GFB X 392      11.576   5.895  10.348  0.00  0.00
ATOM     21  P2  GFB X 392       9.984   6.352  10.839  0.00  0.00
ATOM     22  O9  GFB X 392       9.811   7.841  10.553  0.00  0.00
ATOM     23  O10 GFB X 392       9.036   5.600   9.811  0.00  0.00
ATOM     24  O11 GFB X 392       9.781   5.880  12.234  0.00  0.00
ATOM     25  O4  GFB X 392      12.712   8.039   9.881  0.00  0.00
ATOM     26  C1  GFB X 392      13.263   8.184  11.170  0.00  0.00
ATOM     27  H4  GFB X 392      12.696   9.014  11.554  0.00  0.00
ATOM     28  H8  GFB X 392      13.062   7.292  11.729  0.00  0.00
ATOM     29  C1  0fB X 393       8.548   8.921   8.606  0.00  0.00
ATOM     30  H1  0fB X 393       8.299   7.887   8.783  0.00  0.00
ATOM     31  C2  0fB X 393       9.907   9.481   8.187  0.00  0.00
ATOM     32  H2  0fB X 393      10.475   9.451   9.144  0.00  0.00
ATOM     33  C3  0fB X 393       9.872  10.902   7.674  0.00  0.00
ATOM     34  H3  0fB X 393       9.290  10.810   6.749  0.00  0.00
ATOM     35  C4  0fB X 393       9.063  11.804   8.775  0.00  0.00
ATOM     36  H4  0fB X 393       9.008  12.859   8.329  0.00  0.00
ATOM     37  C5  0fB X 393       7.699  11.276   9.100  0.00  0.00
ATOM     38  H5  0fB X 393       7.020  11.333   8.233  0.00  0.00
ATOM     39  C6  0fB X 393       7.044  11.927  10.338  0.00  0.00
ATOM     40  H61 0fB X 393       6.709  12.975  10.058  0.00  0.00
```

```
ATOM     41  H62 0fB X 393       6.217  11.318  10.711  0.00  0.00
ATOM     42  H63 0fB X 393       7.848  12.128  11.100  0.00  0.00
ATOM     43  O5  0fB X 393       7.759   9.758   9.268  0.00  0.00
ATOM     44  O4  0fB X 393      10.011  11.816   9.864  0.00  0.00
ATOM     45  H4O 0fB X 393       9.899  12.366  10.724  0.00  0.00
ATOM     46  O3  0fB X 393      11.139  11.458   7.378  0.00  0.00
ATOM     47  H3O 0fB X 393      11.226  11.422   6.366  0.00  0.00
ATOM     48  O2  0fB X 393      10.432   8.793   7.102  0.00  0.00
ATOM     49  H2O 0fB X 393      10.915   7.937   7.407  0.00  0.00
ATOM     50  O   WAT X5021       6.287   4.054  10.064  0.00  0.00
ATOM     51  H1  WAT X5021       5.972   4.184  10.993  0.00  0.00
ATOM     52  H2  WAT X5021       7.103   4.601   9.993  0.00  0.00
ATOM     53  DUM DUM X   5      14.305   8.337  11.325  0.00  0.00
ATOM     54  DUM DUM X   5       6.281   9.338   5.171  0.00  0.00
ATOM     55  DUM DUM X   5      10.495   4.047   5.501  0.00  0.00
END
```

Products state

```
ATOM      1  CB  ASN X  21      10.268   4.961   4.672  0.00  0.00
ATOM      2  HB2 ASN X  21      11.119   5.635   4.832  0.00  0.00
ATOM      3  HB3 ASN X  21       9.767   4.963   3.598  0.00  0.00
ATOM      4  CG  ASN X  21       9.276   5.533   5.691  0.00  0.00
ATOM      5  OD1 ASN X  21       8.676   6.634   5.178  0.00  0.00
ATOM      6  ND2 ASN X  21       8.976   5.194   6.937  0.00  0.00
ATOM      7 HD21 ASN X  21       9.257   5.756   8.589  0.00  0.00
ATOM      8 HD22 ASN X  21       9.309   4.209   7.225  0.00  0.00
ATOM      9  CB  THR A 366       6.296   9.219   6.120  0.00  0.00
ATOM     10  HB  THR A 366       5.820   9.935   6.865  0.00  0.00
ATOM     11  CG2 THR A 366       5.591   7.883   6.169  0.00  0.00
ATOM     12 HG21 THR A 366       5.866   7.155   5.425  0.00  0.00
ATOM     13 HG22 THR A 366       5.681   7.293   7.121  0.00  0.00
ATOM     14 HG23 THR A 366       4.476   8.020   6.105  0.00  0.00
ATOM     15  OG1 THR A 366       7.773   9.172   6.458  0.00  0.00
ATOM     16  HG1 THR A 366       8.183   7.231   5.804  0.00  0.00
ATOM     17  O1  GFB X 392      13.984   5.836   9.248  0.00  0.00
ATOM     18  P1  GFB X 392      12.658   6.508   9.091  0.00  0.00
ATOM     19  O2  GFB X 392      12.028   6.688   7.784  0.00  0.00
ATOM     20  O3  GFB X 392      11.607   5.768  10.115  0.00  0.00
ATOM     21  P2  GFB X 392      10.106   6.212  10.731  0.00  0.00
ATOM     22  O9  GFB X 392      10.012   7.643  11.185  0.00  0.00
ATOM     23  O10 GFB X 392       9.140   6.297   9.443  0.00  0.00
ATOM     24  O11 GFB X 392       9.868   5.025  11.650  0.00  0.00
ATOM     25  O4  GFB X 392      12.749   7.958   9.797  0.00  0.00
ATOM     26  C1  GFB X 392      13.348   8.168  11.086  0.00  0.00
ATOM     27  H4  GFB X 392      12.566   8.738  11.664  0.00  0.00
ATOM     28  H8  GFB X 392      13.532   7.168  11.518  0.00  0.00
ATOM     29  C1  0fB X 393       8.343   8.939   7.903  0.00  0.00
ATOM     30  H1  0fB X 393       8.120   7.953   8.298  0.00  0.00
ATOM     31  C2  0fB X 393       9.940   9.284   7.725  0.00  0.00
ATOM     32  H2  0fB X 393      10.228   9.077   8.796  0.00  0.00
ATOM     33  C3  0fB X 393       9.988  10.778   7.434  0.00  0.00
ATOM     34  H3  0fB X 393       9.443  11.062   6.489  0.00  0.00
ATOM     35  C4  0fB X 393       9.401  11.626   8.602  0.00  0.00
```

```
ATOM     36  H4  0fB X 393       9.590  12.674   8.245  0.00  0.00
ATOM     37  C5  0fB X 393       7.875  11.238   8.623  0.00  0.00
ATOM     38  H5  0fB X 393       7.491  11.446   7.616  0.00  0.00
ATOM     39  C6  0fB X 393       7.100  11.767   9.800  0.00  0.00
ATOM     40  H61 0fB X 393       6.954  12.825   9.776  0.00  0.00
ATOM     41  H62 0fB X 393       6.124  11.380  10.089  0.00  0.00
ATOM     42  H63 0fB X 393       7.815  11.534  10.701  0.00  0.00
ATOM     43  O5  0fB X 393       7.753   9.788   8.815  0.00  0.00
ATOM     44  O4  0fB X 393      10.298  11.468   9.757  0.00  0.00
ATOM     45  H4O 0fB X 393      10.086  12.206  10.495  0.00  0.00
ATOM     46  O3  0fB X 393      11.359  11.095   7.111  0.00  0.00
ATOM     47  H3O 0fB X 393      11.335  11.836   6.446  0.00  0.00
ATOM     48  O2  0fB X 393      10.522   8.472   6.778  0.00  0.00
ATOM     49  H2O 0fB X 393      11.051   7.877   7.311  0.00  0.00
ATOM     50  O   WAT X5021       6.364   4.359  10.100  0.00  0.00
ATOM     51  H1  WAT X5021       5.934   4.451  11.016  0.00  0.00
ATOM     52  H2  WAT X5021       6.309   5.325   9.799  0.00  0.00
ATOM     53  DUM DUM X   5      14.303   8.635  11.060  0.00  0.00
ATOM     54  DUM DUM X   5       6.320   9.659   5.114  0.00  0.00
ATOM     55  DUM DUM X   5      10.550   3.931   4.970  0.00  0.00
END
```

5. References

1. Li, Z.; Han, K.; Pak, J. E.; Satkunarajah, M.; Zhou, D.; Rini, J. M., Recognition of EGF-like Domains by the Notch-modifying O-Fucosyltransferase POFUT1. *Nat. Chem. Biol.* **2017,** *13*, 757-763.
2. Chen, V. B.; Arendall, W. B., 3rd; Headd, J. J.; Keedy, D. A.; Immormino, R. M.; Kapral, G. J.; Murray, L. W.; Richardson, J. S.; Richardson, D. C., MolProbity: All-Atom Structure Validation for Macromolecular Crystallography. *Acta Crystallogr. D Biol. Crystallogr.* **2010,** *66*, 12-21.
3. Olsson, M. H. M.; Søndergaard, C. R.; Rostkowski, M.; Jensen, J. H., PROPKA3: Consistent Treatment of Internal and Surface Residues in Empirical pKa Predictions. *J. Chem. Theor. Comput.* **2011,** *7*, 525-537.
4. Case, D. A. e. a., AMBER 2018. *University of California, San Francisco* **2018**.
5. Maier, J. A.; Martinez, C.; Kasavajhala, K.; Wickstrom, L.; Hauser, K. E.; Simmerling, C., ff14SB: Improving the Accuracy of Protein Side Chain and Backbone Parameters from ff99SB. *J. Chem. Theor. Comput.* **2015,** *11*, 3696-713.
6. Kirschner, K. N.; Yongye, A. B.; Tschampel, S. M.; Gonzalez-Outeirino, J.; Daniels, C. R.; Foley, B. L.; Woods, R. J., GLYCAM06: a Generalizable Biomolecular Force Field. Carbohydrates. *J. Comput. Chem.* **2008,** *29*, 622-55.
7. Jorgensen, W. L.; Chandrasekhar, J.; Madura, J. D.; Impey, R. W.; Klein, M. L., Comparison of Simple Potential Functions for Simulating Liquid Water. *J. Chem. Phys.* **1983,** *79*, 926-935.
8. Laio, A.; VandeVondele, J.; Rothlisberger, U., A Hamiltonian Electrostatic Coupling Scheme for Hybrid Car-Parrinello Molecular Dynamics Simulations. *J. Chem. Phys.* **2002,** *116*, 6941-6947.
9. Car, R.; Parrinello, M., Unified Approach for Molecular Dynamics and Density-Functional Theory. *Phys. Rev. Lett.* **1985,** *55*, 2471-2474.
10. Brunk, E.; Rothlisberger, U., Mixed Quantum Mechanical/Molecular Mechanical Molecular Dynamics Simulations of Biological Systems in Ground and Electronically Excited States. *Chem. Rev.* **2015,** *115*, 6217-6263.

11. Troullier, N.; Martins, J. L., Efficient Pseudopotentials for Plane-Wave Calculations. *Phys. Rev. B* **1991,** *43*, 1993-2006.
12. Perdew, J. P.; Burke, K.; Ernzerhof, M., Generalized Gradient Approximation Made Simple. *Phys. Rev. Lett.* **1996,** *77*, 3865-3868.
13. Ardèvol, A.; Rovira, C., Reaction Mechanisms in Carbohydrate-Active Enzymes: Glycoside Hydrolases and Glycosyltransferases. Insights from Ab Initio Quantum Mechanics/Molecular Mechanics Dynamic Simulations. *J. Am. Chem. Soc.* **2015,** *137*, 7528-47.
14. Bilyard, M. K.; Bailey, H. J.; Raich, L.; Gafitescu, M. A.; Machida, T.; Iglesias-Fernandez, J.; Lee, S. S.; Spicer, C. D.; Rovira, C.; Yue, W. W.; Davis, B. G., Palladium-Mediated Enzyme Activation Suggests Multiphase Initiation of Glycogenesis. *Nature* **2018,** *563*, 235-240.
15. Iglesias-Fernandez, J.; Hancock, S. M.; Lee, S. S.; Khan, M.; Kirkpatrick, J.; Oldham, N. J.; McAuley, K.; Fordham-Skelton, A.; Rovira, C.; Davis, B. G., A Front-Face 'S_Ni Synthase' Engineered from a Retaining 'Double-S_N2' Hydrolase. *Nat. Chem. Biol.* **2017,** *13*, 874-881.
16. Nose, S., A molecular Dynamics Method for Simulations in the Canonical Ensemble. *Mol. Phys.* **1984,** *52*, 255-268.
17. Laio, A.; Parrinello, M., Escaping Free-Energy Minima. *Proc. Natl. Acad. Sci. USA* **2002,** *99*, 12562-12566.
18. Ensing, B.; De Vivo, M.; Liu, Z. W.; Moore, P.; Klein, M. L., Metadynamics as a Tool for Exploring Free Energy Landscapes of Chemical Reactions. *Acc. Chem. Res.* **2006,** *39*, 73-81.
19. Barducci, A.; Bonomi, M.; Parrinello, M., Metadynamics. *WIREs Comput. Mol. Sci.* **2011,** *1*, 826-843.
20. Tribello, G. A.; Bonomi, M.; Branduardi, D.; Camilloni, C.; Bussi, G., PLUMED 2: New Feathers for an Old Bird. *Comp. Phys. Commun.* **2014,** *185*, 604-613.
21. Ensing, B.; Laio, A.; Parrinello, M.; Klein, M. L., A Recipe for the Computation of the Free Energy Barrier and the Lowest Free Energy Path of Concerted Reactions. *J Phys Chem B* **2005,** *109*, 6676-87.
22. Garcia-Viloca, M.; Gao, J.; Karplus, M.; Truhlar, D. G., How Enzymes Work: Analysis by Modern Rate Theory and Computer Simulations. *Science* **2004,** *303*, 186-195.
23. Humphrey, W.; Dalke, A.; Schulten, K., VMD: Visual Molecular Dynamics. *J. Mol. Graph.* **1996,** *14*, 33-38.
24. Wang, Y.; Spellman, M. W., Purification and Characterization of a GDP-Fucose:Polypeptide Fucosyltransferase from Chinese Hamster Ovary Cells. *J. Biol. Chem.* **1998,** *273*, 8112-8.
25. Wang, Y.; Shao, L.; Shi, S.; Harris, R. J.; Spellman, M. W.; Stanley, P.; Haltiwanger, R. S., Modification of Epidermal Growth Factor-Like Repeats with O-fucose. Molecular Cloning and Expression of a Novel GDP-fucose Protein O-fucosyltransferase. *J. Biol. Chem.* **2001,** *276*, 40338-45.
26. Lira-Navarrete, E.; Valero-Gonzalez, J.; Villanueva, R.; Martinez-Julvez, M.; Tejero, T.; Merino, P.; Panjikar, S.; Hurtado-Guerrero, R., Structural Insights into the Mechanism of Protein O-fucosylation. *PLoS One* **2011,** *6*, e25365.
27. Takeuchi, H.; Wong, D.; Schneider, M.; Freeze, H. H.; Takeuchi, M.; Berardinelli, S. J.; Ito, A.; Lee, H.; Nelson, S. F.; Haltiwanger, R. S., Variant in human POFUT1 Reduces Enzymatic Activity and Likely Causes a Recessive Microcephaly, Global Developmental Delay with Cardiac and Vascular Features. *Glycobiology* **2018,** *28*, 276-283.
28. Huynh, K.; Partch, C. L., Analysis of Protein Stability and Ligand Interactions by Thermal Shift Assay. *Curr. Protoc. Protein. Sc.i* **2015,** *79*, 28.9.1-28.9.14.
